# Construction and analysis of a modular model of caspase activation in apoptosis

**DOI:** 10.1186/1742-4682-5-26

**Published:** 2008-12-10

**Authors:** Heather A Harrington, Kenneth L Ho, Samik Ghosh, KC Tung

**Affiliations:** 1 Department of Mathematics, Imperial College London, London, SW7 2AZ, UK; 2 Centre for Integrative Systems Biology at Imperial College (CISBIC), Imperial College London, London, SW7 2AZ, UK; 3 Courant Institute of Mathematical Sciences, New York University, 251 Mercer Street, New York, NY 10012, USA; 4 The Systems Biology Institute (SBI) 6-31-15 Jingumae M31 6A, Shibuya, Tokyo 150-0001, Japan; 5 Department of Molecular Biophysics University of Texas Southwestern Medical Center, Dallas, TX 75235, USA

## Abstract

**Background:**

A key physiological mechanism employed by multicellular organisms is apoptosis, or programmed cell death. Apoptosis is triggered by the activation of caspases in response to both extracellular (extrinsic) and intracellular (intrinsic) signals. The extrinsic and intrinsic pathways are characterized by the formation of the death-inducing signaling complex (DISC) and the apoptosome, respectively; both the DISC and the apoptosome are oligomers with complex formation dynamics. Additionally, the extrinsic and intrinsic pathways are coupled through the mitochondrial apoptosis-induced channel via the Bcl-2 family of proteins.

**Results:**

A model of caspase activation is constructed and analyzed. The apoptosis signaling network is simplified through modularization methodologies and equilibrium abstractions for three functional modules. The mathematical model is composed of a system of ordinary differential equations which is numerically solved. Multiple linear regression analysis investigates the role of each module and reduced models are constructed to identify key contributions of the extrinsic and intrinsic pathways in triggering apoptosis for different cell lines.

**Conclusion:**

Through linear regression techniques, we identified the feedbacks, dissociation of complexes, and negative regulators as the key components in apoptosis. The analysis and reduced models for our model formulation reveal that the chosen cell lines predominately exhibit strong extrinsic caspase, typical of type I cell, behavior. Furthermore, under the simplified model framework, the selected cells lines exhibit different modes by which caspase activation may occur. Finally the proposed modularized model of apoptosis may generalize behavior for additional cells and tissues, specifically identifying and predicting components responsible for the transition from type I to type II cell behavior.

## Background

Apoptosis, or programmed cell death, is a highly regulated cell death mechanism involved in many physiological processes including development, elimination of damaged cells, and immune response [1-9]. Dysregulation of apoptosis is associated with pathological conditions such as developmental defects, neurodegenerative disorders, autoimmune disorders, and tumorigenesis [10-16]. The apoptotic pathway is characterized by complex interactions of a large number of molecular components which are involved in the induction and execution of apoptosis. Although scientists do not fully understand the entire pathway, key characteristics have been identified which motivates further study of this cellular process.

**Figure 1:**
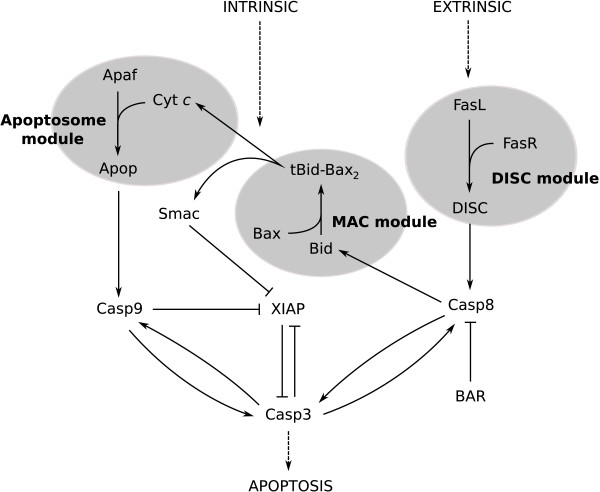
**Extrinsic and intrinsic pathways to caspase-3 activation**. Overview of pathways to caspase-3 activation. Each separate gray region represent the three modules: DISC (death-inducing signaling complex), MAC (mitochondrial apoptosis-induced channel) and apoptosome. Species and their symbols are: FasL (FasL), FasR (FasR), DISC (DISC), procaspase-8 and caspase-8 (Casp8), bifunctional apoptosis inhibitor (BAR), procaspase-3 and caspase-3 (Casp3), XIAP (XIAP), Bid and truncated Bid (Bid), Bax (*Bax*), *tBid *- *Bax*_2 _complex (*tBid *- *Bax*_2_), Smac (Smac), Apaf-1 (Apaf), cytochrome *c *(Cyt*c*), apoptosome (Apop), procaspase-9 and caspase-9 (Casp9). Arrows denote chemical conversions or catalyzed reactions while hammerheads represent inhibition.

As summarized in Figure 1, apoptosis is a cell suicide mechanism in which cell death is mediated by apoptotic complexes along one of two pathways: the extrinsic pathway (receptor mediated) via the death inducing signaling complex (DISC), or the intrinsic pathway (mitochondrial) via the apoptosome [1,17-23].

The extrinsic initiator caspase (caspase-8) couples the two pathways by initiating the mitochondrial apoptosis-induced channel (MAC), leading to the activation of the intrinsic pathway [24]. The subsequent cell death for either pathway is executed through a cascade activation of effector caspases (e.g., caspase-3) by initiator caspases (e.g., caspase-8 and -9) and the amplification of death signals implemented by several positive feedback loops and inhibitors in the network [4,15,16,25,28].

The DISC is formed by the ligation of transmembrane death receptors such as Tumor Necrosis Factor (TNF) Receptor family TNFR1 (CD95, Fas or APO-1) with extracellular death ligands (such as FasL) which cluster and bind to FADD adaptor proteins [21,29-36]. The ensuing complex recruits procaspase-8 through proximity-induced self-cleavage, which leads to the activation of procaspase-8 to caspase-8 [37-39]. Caspase-8 then activates downstream effector caspases such as caspase-3 to induce apoptosis [17].

The intrinsic pathway is activated by stimuli (such as cellular stress or extrinsic pathway signals) inducing mitochondrial membrane permeabilization, followed by the formation of the apoptosome [40,41]. The apoptosome is a large caspase-activating complex [18-20] that assembles in response to cytochrome *c *released from mitochondria due to physical or chemical stress [22,23]. Cytosolic cytochrome *c *activates Apaf-1 [42,43] which oligomerizes to form the apoptosome, a wheel-like heptamer with angular symmetry [19,44]. The apoptosome recruits and activates procaspase-9 through proteolytic cleavage [20]. Caspase-9 then catalyzes the activation of procaspase-3 [45,46].

These apoptotic pathways also include essential positive and negative regulators. Negative regulators such as bifunctional apoptosis inhibitor (BAR) or inhibitor of apoptosis (XIAP) prevent caspase activation; conversely, Smac (DIABLO) which is a protein released with cytochrome *c *from the mitochondria interacts with inhibitors of apoptosis to promote caspase activation [47-50].

Both the extrinsic and intrinsic pathways may converge at the destruction of the mitochondrial membrane. The extrinsic pathway may activate the intrinsic pathway through a mitochondrial apoptosis-induced channel (MAC) of intracellular signals involving the Bcl-2 protein family, which includes both pro-apoptotic (e.g., Bid, tBid, Bax, Bad, Bcl-xs) and anti-apoptotic (e.g., Bcl-2, Bcl-xL) members [51,52].

Specifically, mitochondrial release of cytochrome *c *is enhanced by truncated Bid [53-55]; upon cleavage by caspase-8, Bid translocates to the outer mitochondrial membrane. The MAC formation requires truncated Bid interaction with Bax, leading to membrane pore formation by Bax oligomerization [24,52,56-59]. Corresponding to the two apoptotic signaling pathways are two types of cells [60,61]: in response to death ligands, cells that require DISC formation for apoptotic death are primarily type I (e.g., T cells and thymocytes) while those that release mitochondrial apoptogenic factors are predominately type II cells (e.g., hepatocytes of Bcl-2 transgenic mice) [60-63].

Mathematical models have been employed recently to gain further insights on the complex regulation of caspase activation in apoptosis [57,64-71]. Most of these models focus on specific components of the full apoptotic machinery. Models by Eissing *et al*. [65] and Legewie *et al*. [66] emphasized only either the extrinsic or intrinsic pathways, respectively. The model of Fussenegger *et al*. [67] implemented both pathways but did not consider the coupling between them; however, Bagci *et al*. [57], Albeck *et al*. [72] and Cui *et al*. [73] modeled the mitochondrial apoptosis-induced channel. Stucki *et al*. [68] modeled only the caspase-3 activation and degradation but none of the aforementioned models closely track the upstream formation dynamics of the DISC and the apoptosome, which have since been modeled in detail by Lai and Jackson [74], and by Nakabayashi and Sasaki [75], respectively. Hua *et al*. [69,70] formulated complete system models that incorporate the differences in type I and II signaling as well as include more species, such as Smac; however not all dynamics (e.g. feedbacks) are included from previous component models [65,66,74,75]. More recently, Okazaki *et al*. [71] formulated a model based on Hua *et al*. of the phenotypic switch from type I and type II apoptotic death, but their model does not incorporate protein synthesis or degradation.

The primary focus of this work is to construct the simplest model of caspase-3 activation featuring the oligomerization kinetics of the DISC, mitochondrial apoptosis-induced channel (MAC) and the apoptosome; the dynamics of the extrinsic and intrinsic caspase subnetworks, as well as the coupling between the extrinsic and intrinsic pathways. To accomplish this, we constructed three independent functional modules [76-79]. These are implemented for the abstraction of oligomerization kinetics that simplify the full system. Analysis of the system generates predictions of key system components; furthermore, reduced models are constructed to validate the analysis for different cell types.

## Methods

### Model formulation

The full reaction network of the model is built from three component subnetworks (see Figure 1): the extrinsic, coupling, and intrinsic subnetworks; and three oligomerization modules (represented by gray areas in Figure 1): the DISC, MAC, and apoptosome modules. Each subnetwork captures a vital part of the full apoptotic reaction network and borrows heavily from previous work [57,65,66,70,71], while each module abstracts the oligomerization kinetics of an apoptotic complex to give a simplified net synthesis function using steady-state results [74,75].

The extrinsic subnetwork follows Eissing *et al*. [65] and captures the dynamics of the extrinsic pathway. The subnetwork contains the species FasL, FasR, DISC, procaspase-8 (Casp8), caspase-8 (Casp8*), procaspase-3 (Casp3), caspase-3 (Casp3*), XIAP, and BAR. The subnetwork is driven by DISC, whose formation dynamics from FasL and FasR are encapsulated by the DISC module using the results of Lai and Jackson [74]. DISC induces the cleavage of Casp8 to Casp8*, which then activates Casp3 to produce Casp3*. Positive feedback between Casp8* and Casp3* is provided by the activation of Casp8 by Casp3*. XIAP and BAR act as regulators by binding to Casp3* and Casp8*, respectively. Furthermore, degradation of XIAP is enhanced by Casp3*.

The extrinsic subnetwork can drive the intrinsic pathway through the coupling subnetwork, which describes the role of Casp8* in inducing mitochondrial membrane permeabilization and triggering the release of cytochrome *c *and Smac. The coupling subnetwork takes after a combination of Bagci *et al*., Hua *et al*., and Okazaki *et al*. [57,70,71], and contains the additional species Bid, tBid, Bax, cytochrome *c *(mitochondrial, Cyt*c*; cytosolic Cytc*), and Smac (mitochondrial, Smac; cytosolic, Smac*). The subnetwork receives input from Casp8*, which cleaves Bid to produce tBid. Bax then dimerizes with tBid to form tBid-Bax_2_, which is taken as a representation of the MAC that controls the release of Cyt*c *and Smac from the mitochondria to produce Cytc* and Smac*, respectively; the formation dynamics of tBid-Bax_2 _are abstracted in the MAC module using similar methods as for the DISC module. Morever, Smac* acts as a regulator by binding to XIAP.

The intrinsic subnetwork follows the intrinsic pathway from the assembly of the apoptosome to the resulting caspase interactions. The oligomerization of the apoptosome is abstracted in the apoptosome module using the results of Nakabayashi and Sasaki [75], while the remainder of the subnetwork is simplified from Legewie *et al*. [66]. Additional species contained in the subnetwork include Apaf-1 (Apaf), apoptosome (Apop), procaspase-9 (Casp9), and caspase-9 (Casp9*). The subnetwork is driven by Cytc*, which binds to Apaf; activated Apaf then oligomerizes to form Apop, which cleaves Casp9 to produce Casp9*. As in the extrinsic subnetwork, positive feedback exists between Casp9* and Casp3*. Furthermore, Casp9* binds XIAP.

Constitutive synthesis and degradation rates are assumed for all appropriate species.

### Steady-state abstraction of oligomerization kinetics

The oligomerization kinetics of the DISC, MAC, and the apoptosome are abstracted using steady-state results; this abstraction is a demonstration of a simple technique for modularization and model reduction. For an oligomer *X *with intermediate structures *X*_1_,..., *X*_*n *_and dynamics

$$\frac{d[X]}{dt}=f([X],{\left[X\right]}_{1},...,{\left[X\right]}_{n})-\mu [X],$$

where *f *is the oligomerization rate function and *μ *the degradation rate, use the steady-state approximation *f *≈ *f*_ss _∝ [*X*]_ss_. This allows the modeling of only the final complex and hence significant simplification of the dynamical equations. Although the time dependence of the oligomerization rate is neglected, information regarding the long-term behavior is retained. For the present application, take *f *= [*X*]_ss _with proportionality constant *μ*.

The abstractions for each of the DISC, MAC, and apoptosome modules are described below, where the notation is understood to apply only within each module.

#### DISC module

The DISC oligomerization kinetics are simplified from the crosslinking model [80-82] of Lai and Jackson [74] and follow the reactions

$$\begin{array}{r} \text{FasL}+\text{FasR}\overset{3k_{f}}{\underset{k_{r}}{⇌}}\text{FasL-FasR},\\ \text{FasL-FasR}+\text{FasR}\overset{2k_{f}}{\underset{2k_{r}}{⇌}}{\text{FasL-FasR}}_{2},\\ {\text{FasL-FasR}}_{2}+\text{FasR}\overset{k_{f}}{\underset{3k_{r}}{⇌}}{\text{FasL-FasR}}_{3} \end{array}$$

describing the trimerization of FasR to FasL. With *l *≡ [FasL], *r *≡ [FasR], and *c*_*i *_≡ [FasL-FasR_*i*_], the corresponding dynamics are

$$\begin{array}{cc} \{\begin{array}{l} dl/dt=-v_{1},\\ dr/dt=-v_{1}-v_{2}-v_{3},\\ dc_{1}/dt=v_{1}-v_{2},\\ dc_{2}/dt=v_{2}-v_{3},\\ dc_{3}/dt=v_{3}, \end{array} & \{\begin{array}{l} v_{1}=3k_{f}l_{r}-k_{r}c_{1},\\ v_{2}=2k_{f}c_{1}r-2k_{r}c_{2},\\ v_{3}=k_{f}c_{2}r-3k_{r}c_{3}, \end{array} \end{array}$$

so at steady state,

$$\begin{array}{ccc} c_{1,\text{ss}}=3l_{\text{ss}}\left(\frac{r_{\text{ss}}}{K_{D}}\right), & c_{2,\text{ss}}=3l_{\text{ss}}{\left(\frac{r_{\text{ss}}}{K_{D}}\right)}^{2}, & c_{3,\text{ss}}=l_{\text{ss}}{\left(\frac{r_{\text{ss}}}{K_{D}}\right)}^{3}, \end{array}$$

where *K*_*D *_= *k*_*r*_/*k*_*f*_. Apply the conservation relations

*l*_0 _= *l *+ *c*_1 _+ *c*_2 _+ *c*_2_, *r*_0 _= *r* + *c*_1 _+ 2*c*_2 _+ 3*c*_3_

to obtain

$$l_{\text{ss}}=\frac{l_{0}}{1+3(r_{\text{ss}}/K_{D})+3{\left(r_{\text{ss}}/K_{D}\right)}^{2}+{\left(r_{\text{ss}}/K_{D}\right)}^{3}},$$

where *r*_ss _is given by solving

$$\begin{array}{cc} r_{\text{ss}}^{4}+\alpha r_{\text{ss}}^{3}+\beta r_{\text{ss}}^{2}+\gamma r_{\text{ss}}-K_{D}^{3}r_{0}=0, & \{\begin{array}{l} \alpha =3l_{0}-r_{0}+3K_{D},\\ \beta =3K_{D}(2l_{0}-r_{0}+K_{D}),\\ \gamma =K_{D}^{2}(3l_{0}-3r_{0}+K_{D}), \end{array} \end{array}$$

which has at most one positive root. Assume now that FADD is in excess (see, e.g., [70,71]) to obtain

[DISC]_ss _= *c*_2,ss _+ *c*_3,ss _≡ *f *(*l*_0_, *r*_0_; *K*_*D*_),

where it is assumed that both FasL-FasR_2 _and FasL-FasR_3 _can propagate the death signal [74]. Externally, in the full reaction network, the oligomerization rate function will be called as *f*_DISC _([FasL]_0_, [FasR]_0_; *K*_DISC_). This abstraction reduces the order of the system by four.

#### MAC module

The oligomerization kinetics of the MAC module are assumed to follow a similar crosslinking model and therefore obey the reactions

$$\begin{array}{cc} \text{tBid}+\text{Bax}\overset{2k_{f}}{\underset{k_{r}}{⇌}}\text{tBid-Bax}, & \text{tBid-Bax}+\text{Bax}\overset{k_{f}}{\underset{2k_{r}}{⇌}}{\text{tBid-Bax}}_{2}. \end{array}$$

With the analogous notation *l *≡ [tBid], *r *≡ [Bax], and *c*_*i *_≡ [tBid-Bax_*i*_], the dynamics are

$$\begin{array}{cc} \{\begin{array}{l} dl/dt=-v_{1},\\ dr/dt=-v_{1}-v_{2},\\ dc_{1}/dt=v_{1}-v_{2},\\ dc_{2}/dt=v_{2}, \end{array} & \{\begin{array}{l} v_{1}=2k_{f}l_{r}-k_{r}c_{1},\\ v_{2}=k_{f}c_{1}r-2k_{r}c_{2}, \end{array} \end{array}$$

so

$$\begin{array}{cc} c_{1,\text{ss}}=2l_{\text{ss}}\left(\frac{r_{\text{ss}}}{K_{D}}\right), & c_{2,\text{ss}}=l_{\text{ss}}{\left(\frac{r_{\text{ss}}}{K_{D}}\right)}^{2}, \end{array}$$

Similar conservation relations then give that

$$l_{\text{ss}}=\frac{l_{0}}{{\left(1+r_{\text{ss}}/K_{D}\right)}^{2}}$$

with

$$\begin{array}{cc} r_{\text{ss}}^{3}+\alpha r_{\text{ss}}^{2}+\beta r_{\text{ss}}-K_{D}^{2}r_{0}=0, & \{\begin{array}{l} \alpha =2l_{0}-r_{0}+2K_{D},\\ \beta =K_{D}(2l_{0}-2r_{0}+K_{D}), \end{array} \end{array}$$

which again has at most one positive root. Therefore,

[tBid-Bax_2_]_ss _= *c*_2,ss _≡ *f *(*l*_0_, *r*_0_; *K*_*D*_),

and externally this will be denoted by $f_{{\text{tBid-Bax}}_{2}}([tBid],{\left[Bax\right]}_{0};K_{tBid-Bax_{2}})$, where the dynamical concentration of tBid is used as input. The abstraction reduces the order of the system by three.

#### Apoptosome module

The oligomerization kinetics of the apoptosome follow the model of Nakabayashi and Sasaki [75] with no dissociation, which considers bimolecular interactions of the form

$$\begin{array}{c} \text{Apaf}+\text{Cyt}c^{*}\overset{k_{1}}{\to }\text{Apaf-Cyt}c^{*},\\ \begin{array}{cc} {\left(\text{Apaf-Cyt}c^{*}\right)}_{i}+{\left(\text{Apaf-Cyt}c^{*}\right)}_{j}\overset{k_{2}}{\to }{\left(\text{Apaf-Cyt}c^{*}\right)}_{k}, & i+j=k\leq 7, \end{array} \end{array}$$

where Apop ≡ (Apaf-Cytc*)_7_. With the nondimensionalizations

$$\begin{array}{ccc} c\equiv \frac{\left[\text{Cyt}c^{*}\right]}{{\left[\text{Apaf}\right]}_{0}}, & a\equiv \frac{\left[\text{Apaf}\right]}{{\left[\text{Apaf}\right]}_{0}}, & x_{i}\equiv \frac{\left[{\left(\text{Apaf-Cyt}c^{*}\right)}_{i}\right]}{{\left[\text{Apaf}\right]}_{0}}, \end{array}$$

the dynamics are

$$\begin{array}{l} \frac{da}{d\tau }=\frac{dc}{d\tau }=-ac,\\ \frac{dx_{1}}{d\tau }=ac-\lambda x_{1}(2x_{1}+x_{2}+\cdots +x_{6}),\\ \begin{array}{cc} \frac{dx_{i}}{d\tau }=\lambda \left[\sum_{j=1}^{\left\lfloor i/2\right\rfloor }x_{j}x_{i-j}-x_{i}\sum_{j=1}^{7-i}(1+\delta_{ij})x_{j}\right], & i=2,\ldots ,7, \end{array} \end{array}$$

where *τ *= *αa*_0_*t*, *λ *= *k*_2_/*k*_1_, and *δ *is the Kronecker delta. Integration of this system until steady state over a range of *c*_0 _generates a curve for *x*_7 _that may be accurately fit with a piecewise exponential function

$$\begin{array}{cc} g(c_{0})=\{\begin{array}{cc} g_{1}(c_{0}), & c_{0}\leq 1\\ g_{2}(c_{0}), & c_{0}>1, \end{array} & g_{i}(c_{0})=\alpha_{i}e^{\beta_{i}c_{0}}+\gamma_{i}, \end{array}$$

Continuity at *c*_0 _= 1 and boundary conditions at *c*_0 _= 0 and ∞ give

$$\begin{array}{cc} g_{1}(c_{0})=\left(\frac{e^{\beta_{1}c_{0}}-1}{e^{\beta_{1}}-1}\right)x_{7,\text{ss}}(1), & g_{2}(c_{0})=[x_{7,\text{ss}}(1)-x_{7,\text{ss}}(\infty )]e^{\beta_{2}(c_{0}-1)}+x_{7,\text{ss}}(\infty ), \end{array}$$

where *β*_1 _and *β*_2 _may be fit for any prescribed *λ*. The apoptosome oligomerzation rate function is then *f*(*c*_0_; *λ*) = *a*_0_*g*(*c*_0_; *λ*), and externally this is *f*_Apop_([Cytc*]/[Apaf]_0_; *λ*_Apop_). This abstraction reduces the order of the system by eight.

#### Remarks on modularization

**Figure 2:**
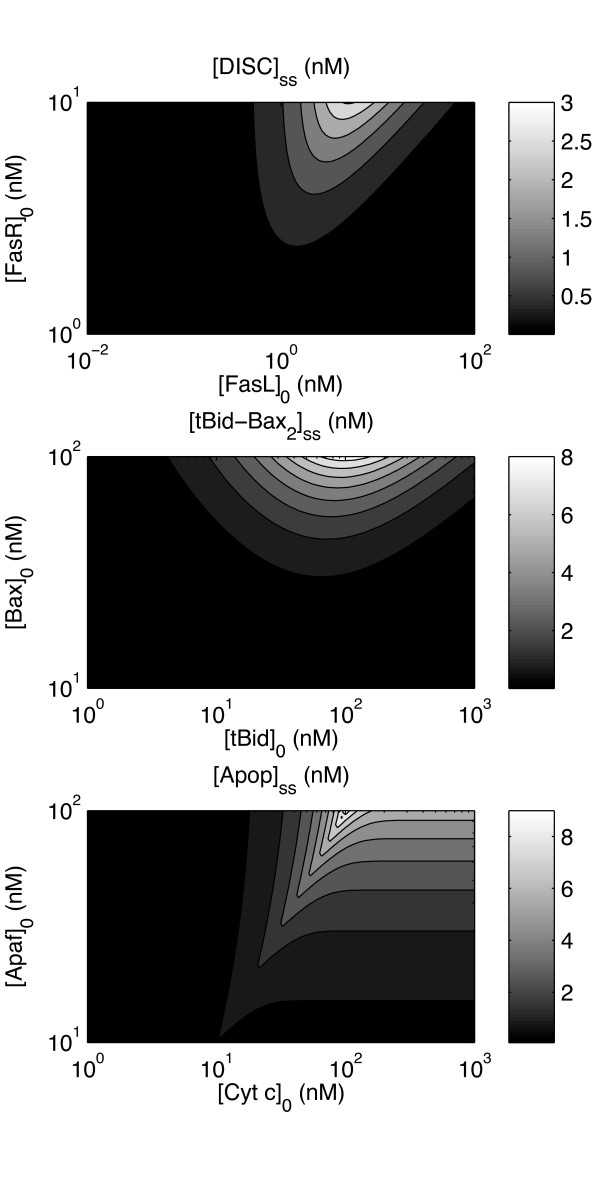
**Steady-state profiles of DISC, tBid-Bax_2_, and apoptosome**. Steady-state concentrations of DISC, tBid-Bax_2_, and apoptosome, used for modularization of the DISC, MAC, and apoptosome modules, respectively. (a) The steady-state DISC concentration [DISC]_ss _as a function of the initial death ligand ([FasL]_0_) and receptor ([FasR]_0_) concentrations. (b) The steady-state tBid-Bax_2 _concentration [tBid-Bax_2_]_ss _as a function of the initial Bax ([Bax]_0_) and tBid ([tBid]_0_) concentrations. (c) The steady-state apoptosome concentration [Apop]_ss _as a function of the initial Apaf-1 ([Apaf]_0_) and cytochrome *c *([Cyt*c*]_0_) concentrations.

The steady-state profiles of the oligomerization kinetics (as shown in Figure 2) are supported by the models that motivated this simplification [74,75] and experimentally for tBid inducing a switch [49]. The abstraction enables these module simplifications to operate as inputs into the full dynamical system of apoptosis.

### Model dynamical equations

The model species and reactions are summarized in Tables 1 and 2. Reaction kinetics are described by mass action, with the corresponding ordinary differential equation (ODE) system given in Table 3. Initial conditions to solve the ODEs for HeLa cells (from [65]) and Jurkat T cells (based on [70,71]), as well as steady-state abstraction parameters, are given in Table 4, where in particular the baseline value of [FasL]_0 _= 2 nM corresponds to a dose which has been used to experimentally induce apoptosis (see [70]).

**Table 1:** Species description, synthesis and degradation rates for the model equations

Species	Description	Synthesis rate (nM/s)	Degradation rate (s^-1^)
DISC	DISC		8.807 × 10^-3^
Casp8	procaspase-8	adjusted	6.5 × 10^-5 ^[65]
Casp8*	caspase-8		9.667 × 10^-5 ^[65]
Casp3	procaspase-3	adjusted	6.5 × 10^-5 ^[65]
Casp3*	caspase-3		9.667 × 10^-5 ^[65]
XIAP	XIAP	adjusted	1.933 × 10^-4 ^[65]
Casp3*-XIAP	Casp3*-XIAP complex		2.883 × 10^-4 ^[65]
BAR	BAR	1.111 × 10^-3^ ([BAR]_0 _= 66.67 nM [65])	1.667 × 10^-5 ^[65]
Casp8*-BAR	Casp8*-BAR complex		1.933 × 10^-4 ^[65]
Bid	Bid	4.168 × 10^-4 ^([Bid]_0 _= 25 nM [70,71])	1.667 × 10^-5 ^(*μ*_BAR_)
tBid	truncated Bid		1.667 × 10^-5 ^(*μ*_Bid_)
tBid-Bax_2_	tBid-Bax_2 _complex		0.0264
Cyt*c*	cytochrome *c *(mitochondrial)	10^-3 ^([Cyt*c*]_0 _= 100 nM [70,71])	10^-5^
Cytc*	cytochrome *c *(cytosolic)		10^-5^
Smac	Smac (mitochondrial)	0.0167 ([Smac]_0 _= 100 nM [70,71])	1.667 × 10^-5 ^(*μ*_BAR_)
Smac*	Smac (cytosolic)		1.667 × 10^-5 ^(*μ*_Smac_)
Smac*-XIAP	Smac-XIAP complex		1.933 × 10^-4 ^(*μ*_Casp8*-BAR_)
Apop	apoptosome		1.487 × 10^-5^
Casp9	procaspase-9	1.3 × 10^-3 ^([Casp9]_0 _= 20 nM [70,71])	6.5 × 10^-5 ^(*μ*_Casp8_)
Casp9*	caspase-9		9.667 × 10^-5 ^(*μ*_Casp8*_)
Casp9*-XIAP	Casp9*-XIAP complex		2.883 × 10^-4 ^(*μ*_Casp3*-XIAP_)

Model species and description are given. In the model, synthesis and degradation rates are given for the model system and labeled *α *and *μ*, respectively.

**Table 2:** Reactions for the model equations

Number	Reaction	Forward rate (nM^-1 ^s^-1^)	Reverse rate (s^-1^)
DISC	(FasL, FasR) → DISC	*f*_DISC_	
1	DISC + Casp8 → DISC + Casp8*	10^-4 ^(*k*_2_)	
2	Casp3* + Casp8 → Casp3* + Casp8*	10^-4 ^[65]	
3	Casp8* + Casp3 → Casp8* + Casp3*	5.8 × 10^-4 ^[65]	
4	Casp3* + XIAP ⇌ Casp3*-XIAP	3 × 10^-3 ^[65]	0.035 [65]
5	Casp3* + XIAP → Casp3*	3 × 10^-3 ^[65]	
6	Casp8* + BAR ⇌ Casp8*-BAR	5 × 10^-3 ^[65]	0.035 [65]
7	Casp8* + Bid → Casp8* + tBid	5 × 10^-4 ^(est. [70,71])	
tBid-Bax_2_	(tBid, Bax) → tBid-Bax_2_	$f_{{\text{tBid-Bax}}_{2}}$	
8	tBid-Bax_2 _+ Cyt*c *→ tBid-Bax_2 _+ Cytc*	10^-3 ^[70,71]	
9	tBid-Bax_2 _+ Smac → tBid-Bax_2 _+ Smac*	10^-3 ^[70,71]	
10	Smac* + XIAP ⇌ Smac*-XIAP	7 × 10^-3 ^[70,71]	2.21 × 10^-3 ^[70,71]
Apop	(Cytc*; Apaf) → Apop	*f*_Apop_	
11	Apop + Casp9 → Apop + Casp9*	2 × 10^-4 ^(est. [66])	
12	Casp3* + Casp9 → Casp3* + Casp9*	2 × 10^-4 ^[66]	
13	Casp9* + Casp3 → Casp9* + Casp3*	5 × 10^-5 ^[66]	
14	Casp9* + XIAP ⇌ Casp9*-XIAP	1.06 × 10^-4 ^[70,71]	10^-3 ^[70,71]

Each reaction described highlights whether the reaction is a forward or reversible reaction by the arrows. The rates are provided from previous work. Reaction are illustrated in Figure 1.

**Table 3:** Ordinary differential equation system for the model

Differential equations	Reaction velocities
*d *[DISC]/*dt *= *μ*_DISC_(*f*_DISC_([FasL]_0_, [FasR]_0_; *K*_DISC_) - [DISC])	*v*_1 _= *k*_1 _[DISC] [Casp8]
*d *[Casp8]/*dt *= -*v*_1 _- *v*_2 _+ *α*_Casp8 _- *μ*_Casp8 _[Casp8]	*v*_2 _= *k*_2 _[Casp3*] [Casp8]
*d *[Casp8*]/*dt *= *v*_1 _+ *v*_2 _- *v*_6 _- *μ*_Casp8* _[Casp8*]	*v*_3 _= *k*_3 _[Casp8*] [Casp3]
*d *[Casp3]/*dt *= -*v*_3 _- *v*_13 _+ *α*_Casp3 _- *μ*_Casp3 _[Casp3]	*v*_4 _= *k*_4 _[Casp3*] [XIAP] - *k*_-4 _[Casp3*-XIAP]
*d *[Casp3*]/*dt *= *v*_3 _- *v*_4 _+ *v*_13 _- *μ*_Casp3* _[Casp3*]	*v*_5 _= *k*_5 _[Casp3*] [XIAP]
*d *[XIAP]/*dt *= -*v*_4 _- *v*_5 _- *v*_10 _- *v*_14 _+ *α*_XIAP _- *μ*_XIAP _[XIAP]	*v*_6 _= *k*_6 _[Casp8*] [BAR] - *k*_-6 _[Casp8*-BAR]
*d *[Casp3*-XIAP]/*dt *= *v*_4 _- *μ*_Casp3*-XIAP _[Casp3*-XIAP]	*v*_7 _= *k*_7 _[Casp8*] [Bid]
*d *[BAR]/*dt *= -*v*_6 _+ *α*_BAR _- *μ*_BAR _[BAR]	*v*_8 _= *k*_8 _[tBid-Bax_2_] [Cyt*c*]
*d *[Casp8*-BAR]/*dt *= *v*_6 _- *μ*_Casp8*-BAR _[Casp8*-BAR]	*v*_9 _= *k*_9 _[tBid-Bax_2_] [Smac]
*d *[Bid]/*dt *= -*v*_7 _+ *α*_Bid _- *μ*_Bid _[Bid]	*v*_10 _= *k*_10 _[Smac*] [XIAP] - *k*_-10 _[Smac*-XIAP]
*d *[tBid]/*dt *= *v*_7 _- *μ*_tBid _[tBid]	*v*_11 _= *k*_11 _[Apop] [Casp9]
*d *[tBid-Bax_2_]/*dt *= $\mu_{{\text{tBid-Bax}}_{2}}$	*v*_12 _= *k*_12 _[Casp3*] [Casp9]
( $f_{{\text{tBid-Bax}}_{2}}$ ([tBid], [Bax]_0 _; $K_{{\text{tBid-Bax}}_{2}}$ ) - [tBid-Bax_2_])	*v*_13 _= *k*_13 _[Casp9*] [Casp3]
*d *[Cyt*c*]/*dt *= -*v*_8 _+ *α*_Cytc _- *μ*_Cytc _[Cyt*c*]	*v*_14 _= *k*_14 _[Casp9*] [XIAP] - *k*_-14 _[Casp9*-XIAP]
*d *[Cytc*]/*dt *= *v*_8 _- *μ *Cytc* [Cytc*]	
*d *[Smac]/*dt *= -*v*_9 _+ *α*_Smac _- *μ*_Smac _[Smac]	
*d *[Smac*]/*dt *= *v*_9 _- *v*_10 _- *μ*_Smac_* [Smac*]	
*d *[Smac*-XIAP]/*dt *= *v*_10 _- *μ*_Smac*-XIAP _[Smac*-XIAP]	
*d *[Apop]/*dt *= *μ*_Apop_(*f*_Apop_([Cytc*]/[Apaf]_0 _; *λ*_Apop_) - [Apop])	
*d *[Casp9]/*dt *= -*v*_11 _- *v*_12 _+ *α*_Casp9 _- *μ*_Casp9 _[Casp9]	
*d *[Casp9*]/*dt *= *v*_11 _+ *v*_12 _- *v*_14 _- *μ*_Casp9* _[Casp9*]	
*d *[Casp9*-XIAP] = *v*_14 _- *μ*_Casp9*-XIAP _[Casp9*-XIAP]	

Ordinary differential equations for the full system are given in the left hand column. Corresponding reaction velocities use mass-action kinetics are found in the right hand column.

**Table 4:** Initial conditions for the model variables and oligomerization parameters

	Initial concentration (nM)			
Species	HeLa	Jurkat T	Parameter	Value
Casp8	216.67 [65]	33.33 [70,71]	[FasL]_0_	2 nM [70,71]
Casp3	35 [65]	200 [70,71]	[FasR]_0_	10 nM [70,71]
XIAP	66.67 [65]	30 [70,71]	*K*_DISC_	1.032 nM [70,71]
BAR	66.67 [65]	66.67 [65]	[Bax]_0_	83.33 nM [70,71]
Bid	25 [70,71]	25 [70,71]	$K_{{\text{tBid-Bax}}_{2}}$	100 nM [70,71]
Cyt*c*	100 [70,71]	100 [70,71]	[Apaf]_0_	100 nM [70,71]
Smac	100 [70,71]	100 [70,71]	*λ*_Apop_	1 [75]
Casp9	20 [70,71]	20 [70,71]		

Initial conditions of model variables are given. Some species initial conditions differ between HeLa or Jurkat T cell type. Parameters and values are given for steady-state oligomerization modules.

Table 5 summarizes all model parameters (forward and reverse reactions, synthesis and degradation rates and parameters for the steady-state abstractions). Additionally, a variant of the Jurkat T cell, denoted Jurkat T*, is considered, which has the the same parameter values as Jurkat T but with *k*_2 _= *k*_5 _= *k*_12 _= 0 following Hua *et al*. and Okazaki *et al*. [70,71].

**Table 5:** Summary of all rates and parameters for the system

	Forward rate		Reverse rate		Synthesis rate		Degradation rate		Parameter
1	*k*_1_	15	*k*_-4_	19	*α*_Casp8_	27	*μ*_DISC_	48	[FasL]_0_
2	*K*_2_	16	*k*_-6_	20	*α*_Casp3_	28	*μ*_Casp8_	49	[FasR]_0_
3	*k*_3_	17	*k*_-10_	21	*α*_XIAP_	29	*μ*_Casp8*_	50	*K*_DISC_
4	*k*_4_	18	*k*_-14_	22	*α*_BAR_	30	*μ*_Casp3_	51	[Bax]_0_
5	*k*_5_			23	*α*_Bid_	31	*μ*_Casp3*_	52	$K_{{\text{tBid-Bax}}_{2}}$
6	*k*_6_			24	*α*_Cytc_	32	*μ*_XIAP_	53	[Apaf]_0_
7	*k*_7_			25	*α*_Smac_	33	*μ*_Casp3*-XIAP_	54	*λ*_Apop_
8	*k*_8_			26	*α*_Casp9_	34	*μ*_BAR_		
9	*K*_9_					35	*μ*_Casp8*-*BAR*_		
10	*k*_10_					36	*μ*_Bid_		
11	*k*_11_					37	*μ*_tBid_		
12	*k*_12_					38	$\mu_{{\text{tBid-Bax}}_{2}}$		
13	*k*_13_					39	*μ*_Cytc_		
14	*K*_14_					40	*μ*_Cytc*_		
						41	*μ*_Smac_		
						42	*μ*_Smac*_		
						43	*μ*_Smac*-XIAP_		
						44	*μ*_Apop_		
						45	*μ*_Casp9_		
						46	*μ*_Casp9*_		
						47	*μ*_Casp9*-XIAP_		

The counter on the left hand columns totals the 54 model rates and parameters for the full system. Each subscript for *k*, *α *and *μ *corresponds to its reaction number. The final column are the parameters used in the abstraction of oligomerization kinetics for the three modules.

The model ODEs are implemented in MATLAB R2007a (The MathWorks, Inc., Natick, Mass., USA) and solved using ode15s.

### Regression analysis and model reduction

**Figure 3:**
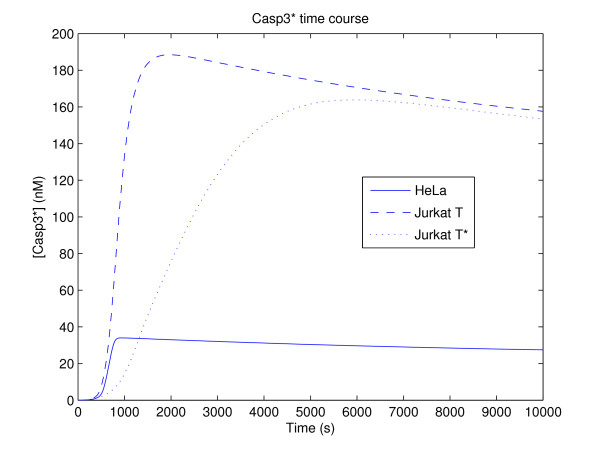
**Caspase-3 time course results**. Time course of caspase-3 activation ([Casp3*]) in HeLa and Jurkat T cells represented by solid and dashed lines, respectively. The time course for a modification of the Jurkat T cell with *k*_2 _= *k*_5 _= *k*_12 _= 0 based on the formulation of Hua *et al*. and Okazaki *et al*. [70,71] is denoted Jurkat T* and represented by the dotted line.

Integration of the model ODEs at baseline parameter values (Table 5) gives the [Casp3*] time courses shown in Figure 3. Both the HeLa and Jurkat T cells (the Jurkat T* case will be addressed in the results) demonstrate a characteristic behavior, whereby [Casp3*] stays low initially, then quickly switches to a high state at some threshold time.

Two quantitative descriptors are used to capture the form of these time courses: the *peak activation*, the maximum value of [Casp3*] attained over the time course; and the *activation time*, the time at which this peak is achieved. To determine the most significant aspects of the model within a given parameter regime, sensitivity analysis is performed with respect to these descriptors according to the following procedure: For a given set of baseline parameter values, we generate normally distributed random parameters about the baseline with standard deviation 5% of the baseline values. Then we simulate the model at these parameters, compute the descriptors and repeat this 100 times (the model has 54 parameters) to collect a set of synthetic data.

Since only local parameter perturbations have been considered, linear relationship ***y ***= (**1*X***)***b ***is assumed between the standardized descriptors ***y ***(***y ***being one of [Casp3*]_max _and *τ *in standardized form) and the standardized random parameters ***X***, where each row of ***X ***is a concatenation of the 54 model parameters in the order given by Table 5. The relation ***b ***is solved by multiple linear regression and large regression coefficients are taken to indicate essential components of the network. This information is used to guide the formulation of reduced models.

## Results and discussion

### Regression analyses and reduced models for FasL induction

**Figure 4:**
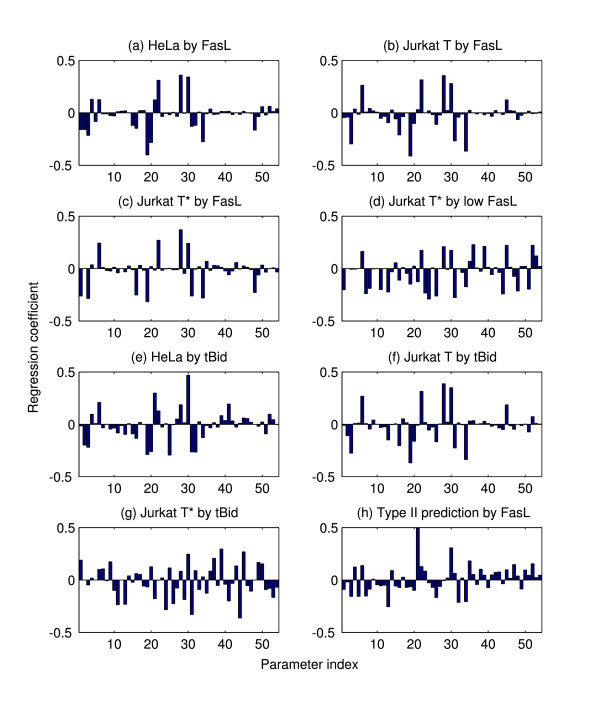
**Regression analysis of apoptosis under various conditions**. Activation time regression coefficients for sample model cases. The activation time is defined as the time at which the peak caspase-3 concentration over the time course occurs. The regression coefficients are ordered by their parameter indices as shown in Table 5. Induction by FasL ([FasL]_0 _= 2 nM unless noted) corresponds to receptor-mediated apoptosis, while induction by tBid corresponds to mitochondrial apoptosis ([tBid]_0 _= 25 nM and [FasL]_0 _= 0 unless otherwise noted). (a) HeLa cell induced by FasL (*r*^2 ^= 0.9958). (b) Jurkat T cell induced by FasL (*r*^2 ^= 0.9903). (c) Jurkat T* cell induced by FasL (*r*^2 ^= 0.9846). (d) Jurkat T* cell induced by low FasL ([FasL]_0 _= 0.01 nM; *r*^2 ^= 0.9569). (e) HeLa cell induced by tBid (*r*^2 ^= 0.9705). (f) Jurkat T cell induced by tBid (*r*^2 ^= 0.9879). (g) Jurkat T* cell induced by tBid (*r*^2 ^= 0.8873). (h) Predicted type II apoptosis cell parameters (*k*_-4 _= *k*_-6 _= 10^-3 ^s^-1^, [XIAP]_0 _= 200 nM, [FasR]_0 _= 1 nM) induced by FasL (*r*^2 ^= 0.9264).

**Figure 5:**
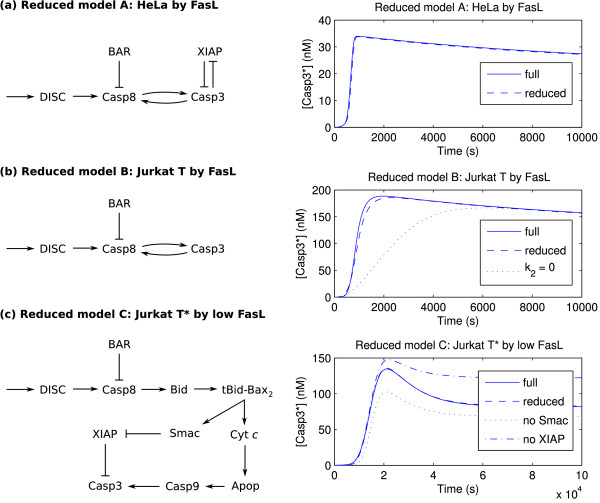
**Reduced models under induction by FasL**. Reduced models of apoptosis under induction by FasL (receptor-mediated apoptosis; [FasL]_0 _= 2 nM unless noted), with time course validations. In (a) and (c), the time courses of the full and reduced models essentially overlap. (a) HeLa cell induced by FasL. (b) Jurkat T cell induced by FasL. (c) Jurkat T* cell induced by low FasL ([FasL]_0 _= 0.01 nM).

Regression analysis as described previously is performed for baseline HeLa parameter values. Regression coefficients for each of the descriptors show isolated peaks, indicating that only a small subset of the network is responsible for the system behavior. Particularly, the coefficients for the peak activation (*r*^2 ^= 0.9991) show strong components only at the synthesis and degradation rates *α*_Casp3 _and *μ*_Casp3_, which together control the initial concentration [Casp3]_0_; evidently, this turns out to largely be the case for all parameter sets considered (not shown), so the peak activation will not be generally further discussed. More interesting is the result for the activation time (*r*^2 ^= 0.9958; see Figure 4a), which, notably, shows that only the reactions of the extrinsic subnetwork appear to be essential. Accordingly, a reduced model (Figure 5a) consisting only of the extrinsic subnetwork is formulated, and validation of the reduction is given by comparison of the [Casp3*] time courses between the full and reduced models.

Note that this result should be expected since the HeLa cell was used in Eissing *et al*. [65] to study type I apoptosis. Surprising, a similar analysis of the Jurkat T cell, whose initial concentration parameters were used to study type II apoptosis by Hua *et al*. and Okazaki *et al*. [70,71], leads to a similar reduction. The regression coefficients (for the activation time; *r*^2 ^= 0.9903) are shown in Figure 4b, with reduction shown in Figure 5b, which is just that for the HeLa case but with XIAP omitted. It should be noted that the regression analysis does not show a strong component at *k*_2_, perhaps due to the corresponding reaction occurring at saturation; therefore not sensitive to small perturbations. Nevertheless, simulations show the necessity to capture the correct dynamics.

Review of the literature reveals that Hua *et al*. and Okazaki *et al*. [70,71] used the model variant denoted as Jurkat T* in this work; for completeness, analysis of the Jurkat T* was hence considered. While induction of the Jurkat T* cell by baseline FasL still shows characteristic type I behavior (Figure 4c, *r*^2 ^= 0.9846; see also the delayed activation in Figure 3), a transition to type II apoptosis is observed for low FasL ([FasL]_0 _= 0.01 nM), in accordance with the transition reported Okazaki *et al*. [71]. This is to be compared against the low FasL cases for the HeLa and Jurkat T cells, which do not exhibit such a transition (not shown). The activation time regression coefficients for the Jurkat T* cell induced by low FasL case are shown in Figure 4d (*r*^2 ^= 0.9569), which in particular has strong components at *k*_7 _and *k*_8_, which describe Bid truncation and the release of Cyt*c*. Moreover, the peak activation regression coefficients (*r*^2 ^= 0.9972, not shown) exhibit a strong contribution by *α*_Smac_. The reduced model (Figure 5c) is correspondingly dominated by the intrinsic pathway; indeed, there is no direct interaction between Casp8 and Casp3 at all. Furthermore, as implicated by the synthesis rate of its inactive form, Smac*, and correspondingly its target XIAP, plays a vital role in achieving the correct activation level, which in particular illustrates the critical role of the shared-inhibitor motif in apoptosis as discussed by Legewie *et al*. [66].

### Regression analyses and reduced models for mitochondrial apoptosis

The behavior of the system pathways under mitochondrial apoptosis can also be studied. Cell stressors that cause the depolarization and permeabilization of the mitochondrial membrane are functionally represented in the model by an input [tBid]_0 _= 25 nM (now [FasL]_0 _= 0). As for the FasL case, peak activation regression coefficients for the cases considered below are dominated by *α*_Casp3 _and *μ*_Casp3_; therefore, will not be further discussed.

**Figure 6:**
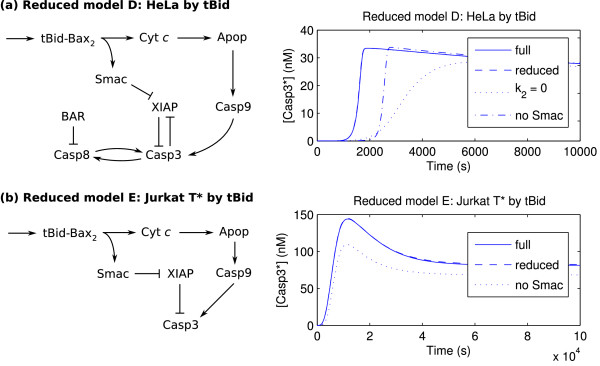
**Reduced models by tBid**. Reduced models of apoptosis under induction by tBid (mitochondrial apoptosis; [tBid] = 25 nM and [FasL]_0 _= 0), with time course validations. In both cases, the time courses of the full and reduced models essentially overlap. (a) HeLa cell induced by tBid. (b) Jurkat T* cell induced by tBid.

Performing the regression analysis on the HeLa cell induced by tBid produces the activation time regression coefficients shown in Figure 4e (*r*^2 ^= 0.9705). Strong components corresponding to the reactions of the intrinsic subnetwork are observed; interestingly, the system behavior is sensitive to several extrinsic reactions as well. The model reduction is shown in Figure 6a, which demonstrates that the extrinsic caspase feedback between Casp8 and Casp3 is essential to capturing the correct dynamics (compare the time course with *k*_2 _= 0). Thus, the HeLa cell displays an apoptotic mechanism that involves the intrinsic pathway triggering the extrinsic pathway. Furthermore, the role of Smac* as an indirect activator of Casp3 through the sequestration of XIAP is recovered. Although Casp9* possesses a similar sequestration ability, the analysis reveals that the primary role of Casp9* is through direct activation of Casp3.

Analysis of the Jurkat T cell induced by tBid gives similar results (Figure 4f, *r*^2 ^= 0.9879; reduced model not shown), though the magnitude of the regression coefficient of *k*_13_, which describes the activation of Casp3 by Casp9*, is larger than in the HeLa case, suggesting a stronger role for the intrinsic caspase. For completeness, the Jurkat T* cell is induced by tBid is also considered. The activation time regression coefficients are shown in Figure 4g. In this case, the fit is relatively poor (*r*^2 ^= 0.8873) and some parameters are selected in error (e.g., *k*_1_, which has no effect on the system by construction; also note the larger number of significant components). Nevertheless, the regression serves to guide the model reduction, which in this case required manual correction. The reduced model (Figure 6b) reveals a purely intrinsic mechanism of caspase activation. Similarly to the HeLa and Jurkat T cells, the sequestration of XIAP by Smac* is essential, while that by Casp9* may be neglected.

Although the peak activation for each of the HeLa, Jurkat T, and Jurkat T* cells is essentially identical to that obtained under FasL induction, the activation time shows a significant increase (factor increase of 2.1457, HeLa; 1.3003, Jurkat T; 1.9920, Jurkat T*). This is in general agreement with experimental evidence that caspase activation through the intrinsic pathway is delayed relative to that through the extrinsic pathway [62].

#### Type II apoptosis prediction

In the preceding cases considered, type II apoptosis was observed only for the Jurkat T* cell under low FasL induction. This may be unsatisfactory since the Jurkat T* cell omits caspase feedback interactions which suggest potentially questionable biological relevance. Thus, a natural idea is to determine whether parameters leading to type II apoptosis may be predicted for the full reaction network rather than resorting to the Jurkat T* formulation.

An attempt to use the regression analysis for this task was made based on the idea of performing regression with respect to differences in the peak activation and in the activation times between a given parameter set and the corresponding set with *k*_7 _= 0 (no Bid truncation, i.e., no extrinsic-intrinsic coupling). The intuition in this analysis is that strong regression coefficients (assuming the modified descriptors are taken with the appropriate sign) now select parameters whose increase may effect a transition from type I to type II behavior. Furthermore, the parameters randomly perturbed are now restricted to only the synthesis and degradation rates, and to [FasL]_0_, [FasR]_0_, [Bax]_0_, and [Apaf]_0_, i.e., the parameters that control only the initial concentration, referred to as *cell-specific parameters*, as these are presumably the only parameters which may vary between different cell types.

Unfortunately, the regression coefficients for this analysis give poor fits (0.3884 ≤ *r*^2 ^≤ 0.7714 for the activation time difference) for the cases considered, so the method fails. However, progress may nevertheless be made by considering the result from the case of Jurkat T* induced by low FasL. The strategy is to transform the conditions of that case into equivalent cell-specific parameter conditions. For example, the Jurkat T* cell mutes the reactions involving the action of Casp3* on other molecules. This effect may be achieved in principle by increasing [XIAP]_0 _and hence the inhibition of Casp3*, which turns out to be insufficient as a result of the strong positive feedback between Casp8 and Casp3. Therefore, it is further necessary to decrease the rate at which Casp8 is activated. This may be controlled at the DISC module, so accordingly decrease [*FasR*]_0 _(for the dependence, see Figure 2a).

**Figure 7:**
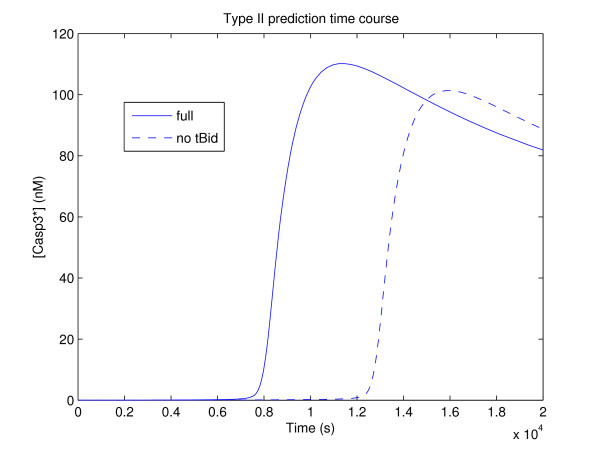
**Type II prediction time course**. Time course of caspase-3 activation ([Casp3*]) for the type II apoptosis cell prediction parameters (*k*_-4 _= *k*_-6 _= 10^-3 ^s^-1^, [XIAP]_0 _= 200 nM, [FasR]_0 _= 1 nM) induced by [FasL]_0 _= 2 nM. The solid line gives the time course of the full model, while the dashed line gives the time course with *k*_7 _= 0 (i.e., no Bid truncation, hence no extrinsic-intrinsic coupling). Note the significant delay in caspase activation.

At the assumed rate parameters, however, the changes in [XIAP]_0 _and [FasR]_0 _required to achieve type II apoptosis are rather dramatic. Note though that the dissociation rates *k*_-4 _and *k*_-6 _of Casp3*-XIAP and Casp8*-BAR, respectively, as estimated from Eissing *et al*. [65] are suspiciously large; if the estimate *k*_-4 _= *k*_-6 _= 10^-3 ^s^-1 ^is taken instead, more consistently with, e.g., [66,70,71], then the changes required are no more than an order of magnitude. Specifically, starting with Jurkat T parameters, increasing [XIAP]_0 _from 20 to 200 nM, and decreasing [FasR]_0 _from 10 to 1 nM gives a cell type for which the intrinsic pathway is significant even under high FasL induction. The sensitivity regression analysis for this cell is shown in Figure 4h (*r*^2 ^= 0.9264), which displays significant components corresponding to the intrinsic subnetwork, notably at *k*_13_. The influence of the intrinsic pathway demonstrated by the comparison of time courses in Figure 7 shows a significant delay of caspase activation upon disabling the pathway coupling through tBid. In comparison, control results for the HeLa and Jurkat T cells show no such dependence (not shown).

Perhaps in light of this result, an alternative interpretation of the fact that the modified regressions produced poor fits occurs due to type II transitions requiring large changes that the local character of the linear regression cannot capture. This is consistent with the changes that Hua *et al*. and Okazaki *et al*. [70,71] report to effect transitions in their models (without caspase feedback), where, effectively, [Casp8]_0 _was modified by a similar amount. As a final note, changes of this magnitude are likely reasonable given the inherent variability experimentally observed (see, e.g., [83]).

### Activation thresholds and stability

**Figure 8:**
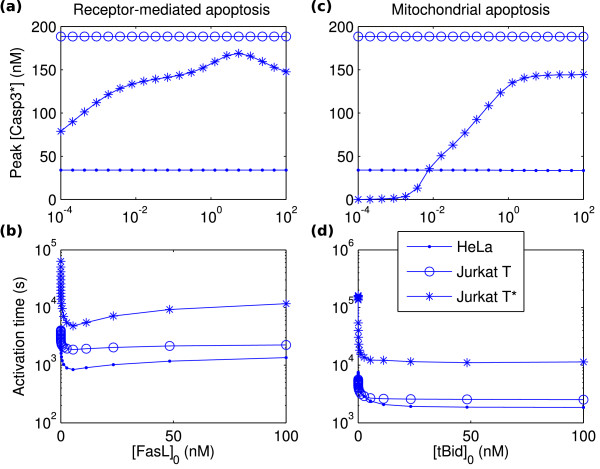
**Peak caspase-3 activations and activation times**. Peak caspase-3 activations and activations times for HeLa (dots), Jurkat T (circles), and Jurkat T*(asterisks) cells under receptor-mediated apoptosis (variable [FasL]_0_) and mitochondrial apoptosis (variable [tBid]_0 _with [FasL]_0 _= 0). (a) Peak activations for receptor-mediated apoptosis. (b) Activation times for receptor-mediated apoptosis. (c) Peak activations for mitochondrial apoptosis. (d) Activation times for mitochondrial apoptosis.

It should be noted that the model in its present formulation is unstable, even to transient signals, as Figure 7 suggests. However, some notion of stability may nevertheless be achieved by considering activation times. This is shown for HeLa, Jurkat T, and Jurkat T* cells in Figure 8.

Consider first the case of receptor-mediated apoptosis, i.e., by FasL induction. For HeLa and Jurkat T cells, the peak activation is essentially constant (Figure 8a) with [FasL]_0_, in accordance with the observation from the regression analyses that the peak activation is relatively insensitive. However, the activation time (Figure 8b) varies significantly, showing first a sharp decrease with [FasL]_0 _for low [FasL]_0_, then a gradual leveling-off as [FasL]_0 _increases thereafter. Clearly, this latter portion may be interpreted as the cell undergoing apoptosis in a saturated manner, in which further increase of the death signal no longer affects the response time. Analogously, the initial drop appears to define a transition region, wherein the cell switches from slow to fast apoptotic dynamics over a narrow range of the death signal input; this is indicative of some threshold-like behavior. Although this is not bistability, a sense of the existence of both low and high apoptotic states is nevertheless furnished, which, furthermore, may be made precise by introducing an artificial cutoff on the activation time to discount activations which take too long to occur. The case of the Jurkat T* cell is similar, though now the peak activation does show nontrivial variation with [FasL]_0_. However, the peak activation remains uniformly rather high which questions biological significance.

Corresponding data for mitochondrial apoptosis (variable [tBid]_0 _with [FasL]_0 _= 0) are shown in Figure 8c, d. The cases for the HeLa and Jurkat T cells are similar as the receptor-mediated case; however, the Jurkat T* cell appears to exhibit bistability (Figure 8c). For low FasL (approximately [FasL]_0 _< 10^-2 ^nM), the peak activation stays low (near zero), whereas for high FasL ([FasL]_0 _> 1 nM), the peak activation reaches a high state around 145 nM. Intermediate concentrations define a transition region where the cell may be interpreted to switch from life to death.

The present data was computed with a constant input for the receptor-mediated case and an exponentially decaying (i.e., transient) input for the mitochondrial case (since tBid has a constitutive degradation rate in the model). Interestingly, instituting a transient FasL signal, with estimated degradation rate *μ*_FasL _= 10^-5 ^s^-1^, gives no discernable change to the receptor-mediated data, while setting *μ*_tBid _= 0 degrades the quality of the bistability result of the Jurkat T* case for mitochondrial apoptosis (not shown). This affords some insight into why the noted bistability is observed: by virtue of the delay of apoptosis incurred by the intrinsic pathway through the necessary activation of the mitochondrial apoptogenic factors and the assembly of the apoptosome (compare Figures 8b and 8d), the intrinsic pathway is able to better filter out transient signals.

With regard to stability, perhaps this implies that further models of apoptosis should be careful to include potentially important regulators such as cFLIP, which inhibits DISC and hence imposes a delay on Casp8 activation [70,71]. Moreover, it may likewise be prudent to expand in full any series of activations occuring sequentially; an example might be the interactions between Bid and Bax to form the MAC, which currently is not mechanistically understood and consequently may be inappropriately abstracted; although in defense of the abstraction, it is experimentally suggested that component levels (such as Apaf-1 or Casp9) may determine how quickly some cells die [83]. Finally, in this view, the steady-state abstractions presented are actually quite unsuitable for the purposes of model stability; however, modulation of the given abstracted dynamics by appropriate time-dependent functions (e.g., by an appropriate Heaviside function) may suffice.

## Conclusion

This study has presented a methodological construction of a straightforward and informative mathematical model of apoptosis. This was done by combining both the extrinsic and intrinsic pathways through the implementation of functional modules and subnetworks motivated by previous models and findings [65-67,69-71,74,75]. The subnetworks, responsible for the activation of Casp3 and ultimately apoptosis, included descriptions of both the extrinsic and intrinsic pathways as well as the coupling between them. Modularization of the oligomerization kinetics of the DISC, MAC, and apoptosome were achieved through the implementation of steady-state abstraction techniques.

Sensitivity analysis by linear regression was used to identify key components of the apoptotic network under various cell conditions. This allowed for the formulation of reduced models to capture only the essential dynamics of the system. Importantly, these reductions allowed the extraction of biological insight and helped clarify the roles of specific molecular components. For example, the model predicts for the parameter regimes considered that Casp9* contributes to the activation of Casp3 by direct catalytic activation rather than through sequestration of their common inhibitor XIAP. Furthermore, the reduced models validated many previous findings, including the critical role of XIAP and the shared-inhibitor motif in mediating apoptosis [66,84-87], as well as the transition from type I to type II apoptosis as the induction of the extrinsic pathway is decreased [70,71]. Finally, the analysis revealed the variety of modes through which caspase activation can be achieved. In the cases considered, caspase activation was observed to occur 1) solely through the extrinsic pathway 2) solely through the intrinsic pathway 3) through the extrinsic triggering the intrinsic pathway and 4) through the intrinsic triggering the extrinsic pathway. Whether cells employ all of these modes is an interesting experimental question, with possibly profound biological significance.

The results of the regression analyses were also used to predict cell parameters (i.e., initial concentrations) that would elicit type II apoptosis, even under high FasL induction, without having to use the Jurkat T* model of Hua *et al*. and Okazaki *et al*. [70,71], which omits important caspase feedback interactions [65,66]. This adheres to the notion of highly conserving the apoptosis pathway [1,6,15], and in principle, achieving both type I and type II apoptosis using the same network. Naturally, the type II cell prediction invites experimental investigation.

Furthermore, remarks on caspase activation thresholds and stability were given. The critical element in achieving bistability in the system (at least to transient signals) appears to be related to whether sufficient delays are included. In particular, this implies the importance of modeling regulators, especially inhibitors, of the system, as well as the correct dynamical description of complex formation. Specifically, for this latter point, the present formulation neglects the time dependence of the oligomerization rate and assumes that the formation of a given final complex proceeds without delay. This, however, does not reflect actual dynamics; for example, the model of apoptosome assembly by Nakabayashi and Sasaki [75] at the parameter values considered in this study exhibits a characteristic time delay on the order of 100 min. A simple improvement is the delayed initiation of the present approximation by an appropriate time. A general theory of oligomerization that gives such a time would be particularly useful. Finally, of special interest is whether the incorporation of such delays can recover the expected type II behavior of the Jurkat T cell while maintaining the type I behavior of the HeLa cell.

Future directions for model refinement include more sophisticated treatment of oligomerization kinetics as described. A more comprehensive procedure for model reduction would also be helpful. The current method of sensitivity analysis is unable to eliminate reactions near saturation; however these cases should intuitively be treatable analytically. Moreover, the implementation of a faithful model exhibiting bistability is of primary biological interest as this would allow the formal definition and investigation of a point of no return in apoptosis. Furthermore, it may be profitable to adapt and apply the model to other cell types, e.g., mature neurons, which have repressed Apaf-1 expression and hence apoptosome formation [88,89]. Extending the presented work to model apoptosis at a cell population level may predict key mechanisms; and perhaps, prove fruitful for understanding drug sensitivity in various cell lines.

The model thus presented serves as a guide for future theoretical and experimental work in analyzing apoptosis and achieves progress toward a full model of this important biological process.

## Competing interests

The authors declare that they have no competing interests.

## Authors' contributions

HAH, SG, KLH and KCT equally contributed in constructing and simplifying the model. HAH and SG conducted analysis and KLH and KCT performed simulations. HAH and KLH prepared the initial drafts of the manuscript.
